# Non-linear Normalization for Non-UMI Single Cell RNA-Seq

**DOI:** 10.3389/fgene.2021.612670

**Published:** 2021-04-09

**Authors:** Zhijin Wu, Kenong Su, Hao Wu

**Affiliations:** ^1^Department of Biostatistics, Brown University, Providence, RI, United States; ^2^Department of Computer Science, Emory University, Atlanta, GA, United States; ^3^Department of Biostatistics and Bioinformatics, Emory University, Atlanta, GA, United States

**Keywords:** scRNA sequencing, single cell, normalization, statistical method, gene expression

## Abstract

Single cell RNA-seq data, like data from other sequencing technology, contain systematic technical noise. Such noise results from a combined effect of unequal efficiencies in the capturing and counting of mRNA molecules, such as extraction/amplification efficiency and sequencing depth. We show that such technical effects are not only cell-specific, but also affect genes differently, thus a simple cell-wise size factor adjustment may not be sufficient. We present a non-linear normalization approach that provides a cell- and gene-specific normalization factor for each gene in each cell. We show that the proposed normalization method (implemented in “SC2P" package) reduces more technical variation than competing methods, without reducing biological variation. When technical effects such as sequencing depths are not balanced between cell populations, SC2P normalization also removes the bias due to uneven technical noise. This method is applicable to scRNA-seq experiments that do not use unique molecular identifier (UMI) thus retain amplification biases.

## 1. Introduction

Single Cell RNA-sequencing (scRNA-seq) has become a widely applied tool to study the diverse and dynamic transcriptional activities among cell populations (Tang et al., 2009). Before the RNA-sequencing technology was applied to query the transcriptomes of individual cells, scientists have used it widely to measure mRNA expression from bulk samples (Mortazavi et al., 2008), in which an average level of RNA expression from a large number (often millions) of cells is obtained. Methods for data processing, including mapping short reads to the reference transcriptome and normalization to account for technical variability in the efficiency of RNA extraction, amplification and counting, evolved along the progress of the sequencing technology. These include simple size factors to adjust for global effects such as sequencing depth, such as widely used count per million (CPM) or reads per million per kilobase (RPKM) for their simplicity (Mortazavi et al., 2008), and more data adaptive trimmed mean of M values (TMM) (Robinson and Oshlack, 2010). Noting that non-linear and inconsistent biases due to gene length and GC-content exist in RNA-seq data, more flexible methods have been proposed, such as the conditional quantile normalization (CQN) (Hansen et al., 2012) and remove unwanted variation (RUV) (Risso et al., 2014).

All normalization methods, explicitly or implicitly, make assumption about characteristics of the data that are expected. For example, in many bulk RNA-seq data sets, assumptions on the lack of global shifts of the distribution of expression are often reasonable. As a result, the changes of the location, scale, or shape of the distribution are attributed to technical effects and removed in normalization (Robinson and Oshlack, 2010; Hansen et al., 2012). scRNA-seq data share many similarities of bulk RNA-seq data, but have their unique characteristics. These include, but are not limited to, the much higher percentage of genes with zero count and generally lower library size (Shapiro et al., 2013). In addition, there is often much greater variability among cells compared to that among bulk samples, because bulk samples measure the average expression from a large population of cells (Wu et al., 2014). Thus, it may no longer be reasonable to assume the lack of global differences, and a direct adaptation of bulk RNA-seq normalization is not optimal, despite its convenience.

The need for specialized normalization is well-recognized. Since the introduction of scRNA-seq, a handful of normalization approaches have been proposed (Lun et al., 2016; Bacher et al., 2017). Most analyses of RNA-seq data at least attempt to address this bias due to sequencing depth or overall mRNA capture efficiency by turning the counts data into counts-per-million (CPM). This practice implicitly assumes a linear relationship between library size and the observed counts. There are several problems with this simple practice. One is that the library size (the total observed count in a sample) may not be a stable statistic to represent the overall counting efficiency in a cell. In bulk RNA-seq, each individual gene accounts for a very small fraction of a sample, thus the library size often captures the overall efficiency including sequencing depth and mRNA extraction efficiency. In scRNA-seq, a few top genes can account for a large fraction of total counts, making the library size sensitive to the variation of these genes, which are not necessarily stable across cells. This problem can be alleviated when one uses a more robust estimate of the size factor, such as using TMM. Another issue with a simple size factor adjustment is that it assumes the impact of the size factor is the same to all genes in the same cell. Bacher et al. (2017) showed that this is not necessarily true, and proposed to normalize genes in several groups. Recognizing that common assumptions on an identical distribution of genes expression may not be reasonable across all cells, normalization based on internal ERCC controls have also been proposed (Ding et al., 2015). However, since the control RNAs are spiked in after RNA extraction, the ERCC controls only capture technical biases in a portion of the sample preparation procedures. Though 96 RNAs are included in the ERCC panel, many of them are at levels too low to be detected, making the number of controls that can be used to capture the systematic bias much lower, thus the biases less reliably estimated.

In this manuscript, we describe a simple but effective normalization procedure that captures the potential non-linear, systematic biases in scRNA-seq data. We consider that a gene's observed count is affected by both its expression level (the biological factor) and the detection efficiency (the technical factors). The technical factors include the quality of cell dissociation, mRNA extraction/amplification efficiency, and sequencing depth. These factors may have different impact across genes. The combined effect of these factors on detection efficiency is the technical bias we aim to estimate and remove. Our procedure takes into account both gene-specific and cell-specific contexts in scRNA-seq data, thus borrows information both from the same gene across cells and from other genes within the same cell to achieve a robust normalization factor.

## 2. Results

### 2.1. Data Sets

We use four scRNA-seq data sets to illustrate the normalization performance. The first is from a type 2 diabetes study of pancreatic islet cells, referred to as “T2D" data hereafter. The T2D data set includes 978 cells, of which 239 are alpha cells (Lawlor et al., 2017). We use the alpha cells as an example to illustrate variation within a cell type. This data set is available at Gene Expression Omnibus (GEO) with accession number GSE86473. The second data set (GEO accession number GSE85917) profiles human embryonic stem cells, referred to as “hESC" data hereafter. There are 92 H1 cells sequenced twice with very different sequencing depth: approximately one and four million reads per cell. This data set was originally generated to evaluate SCnorm normalization method (Bacher et al., 2017). The third data set (GEO accession number GSE45719) profiles cells in different early development stages ranging from zygote to blastocyst and is referred to as the “embryo" data using Smart-seq (Deng et al., 2014). The fourth data set (GEO accession number GSE75748) comes from a time course experiment that measured hESC cells at different time points, including 758 cells, and is referred to as the “time course" data (Chu et al., 2016).

### 2.2. The Technical Bias May Not Be a Constant Linear Effect of Library Size

The impact of overall mRNA extraction efficiency and sequencing depth is well-known. In single cell data this is reflected in two ways: cells with higher library size tend to have higher gene detection rate (the proportion of genes with non-zero count), and tend to have higher counts on the genes that are observed. The simplest adjustment for this overall effect is turning the counts data into counts-per-million (CPM). This practice inexplicitly assumes a linear relationship between library size and the observed counts, and makes the same adjustment for all genes in a given cell. We first demonstrate that technical bias depends on the gene as well, and is not always a simple linear effect.

**Figure 1:**
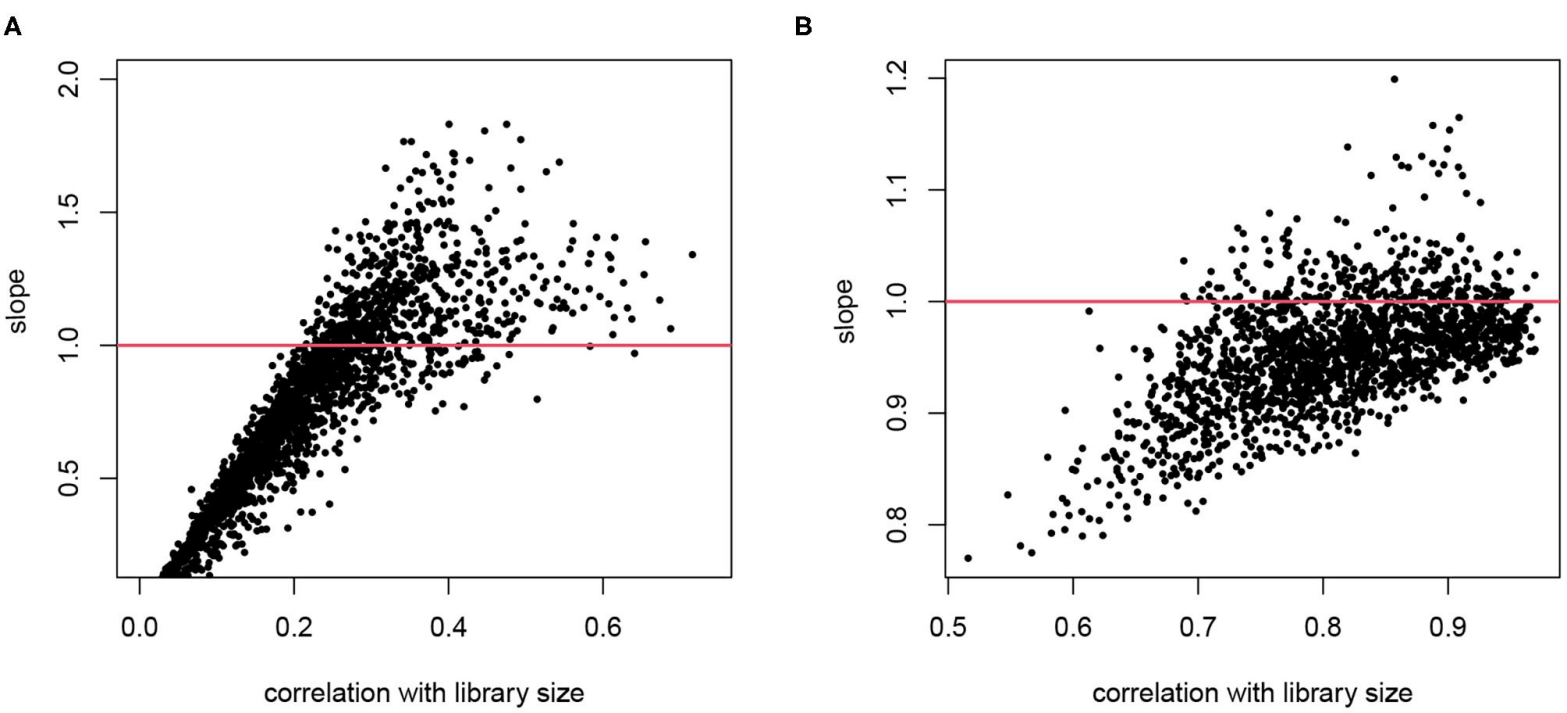
The relationship between counting efficiency with library size. The log transformed counts are regressed on log library size and the slope is plotted against the correlation between the gene counts and the library size. A slope of 1 indicates a linear effect of library size on counting efficiency. **(A)** Data from T2D study. **(B)** Data from hESC study. Only genes with average log counts greater than 4 are included.

For cell *i*, denote the library size by *L*_*i*_. Consider gene *g* in this cell, denote its gene expression level as *θ*_*gi*_, and the observed read count as *Y*_*gi*_. When we assume that *E*[*Y*_*gi*_] ∝ *θ*_*gi*_*L*_*i*_, normalizing by *Y*_*gi*_/*L*_*i*_ is a reasonable practice. This type of normalization, using a cell-wise size factor, implies log(*E*[*Y*_*gi*_]) = log(*θ*_*gi*_)+log(*L*_*i*_)+*c*. It means that the log transformed counts are proportional to log library size with a constant slope 1 for all genes. We explore these assumptions in real scRNA-seq data as shown in Figure 1, where we plot the slope of log counts regressing on library size against the correlation between a gene's counts and library size across cells. If we had a constantly expressed gene with *θ*_*gi*_ ≡ *θ*_*g*_ and the gene counts are proportional to *L*_*i*_, we would have a perfect correlation and slope 1. Here we focus on genes that are reliably detected and only include those with average log counts greater than 4. As expected, the counts for many genes are strongly correlated with library size, confirming that the library size indeed affects measured expression level, though the correlation is lower than 1 since there are natural variations of expression levels even within the same cell type. The correlation with *L*_*i*_ is lower for genes with high biological variation or genes with low expression and hence under greater influence of Poisson counting error. The slopes from genes that are highly correlated with library size are the most informative of the extent of the technical bias. We observe that the assumption of a constant slope of 1 is inaccurate in two senses: (1) the slopes between log(*Y*_*gi*_) and log(*L*_*i*_) are not necessarily the same for all genes; and (2) the slope on average is not necessarily 1. In the T2D data, the slope tends to exceed 1 for genes that show high correlation with library size, whereas in the hESC data the slope tends to be lower.

### 2.3. Not All Genes Reflect Technical Bias in a Cell

Bacher et al. (2017) report similar observations that the need for normalization differs for different genes and give specific examples of genes with high, median and even negative slope in this relationship in the data used in Figure 1B. As a solution, they divide the genes into multiple bins and estimate their “count-depth relationship" separately, and normalize accordingly.

**Figure 2:**
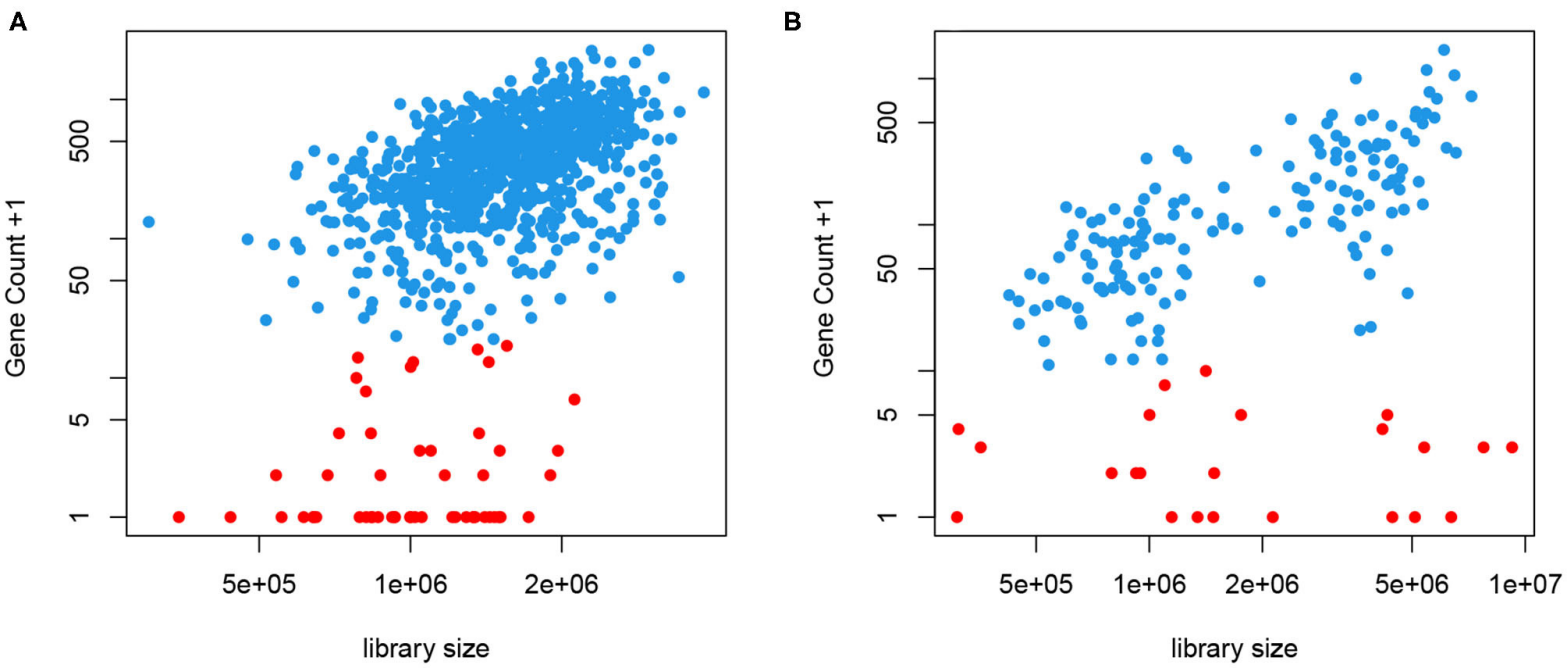
Gene counts are positively correlated with library size when the gene is expressed (blue) but appear to have little correlation with library size when they are in the background phase (red). **(A)** Data from T2D study. **(B)** Data from hESC study.

We take a different approach here without putting genes into bins. Instead, we obtain a cell- and gene-specific normalization factor that depends on the mean expression level, represented by a smooth function. This is motivated by the fact that most, if not all, genes are not transcribed in all cells. When a gene is expressed, we often observe a close-to-linear relationship between the gene count and the library size, as seen in Figure 2. This means that a higher count observed could be a result of higher sequencing depth or higher mRNA extraction success in certain cells, instead of higher expression level. This is the motivation behind CPM type of normalization. However, we also notice that even in cells with very high library size, we often observe low but non-zero counts, shown in red in Figure 2. We have introduced a two-phase expression model, *SC2P*, for scRNA-seq data that account for these two latent phases (Wu et al., 2018). Phase I corresponds to a background level of counts which represent the inactive phase, and Phase II corresponds to the phase when the gene is actively transcribed. For a cell that has high extraction/amplification rate and is sequenced deeply, the active genes in it tend to show higher counts. In the same cell, genes in Phase I will only have a low, background level of counts, regardless of the library size.

### 2.4. Technical Bias Depends on Expression Level

**Figure 3:**
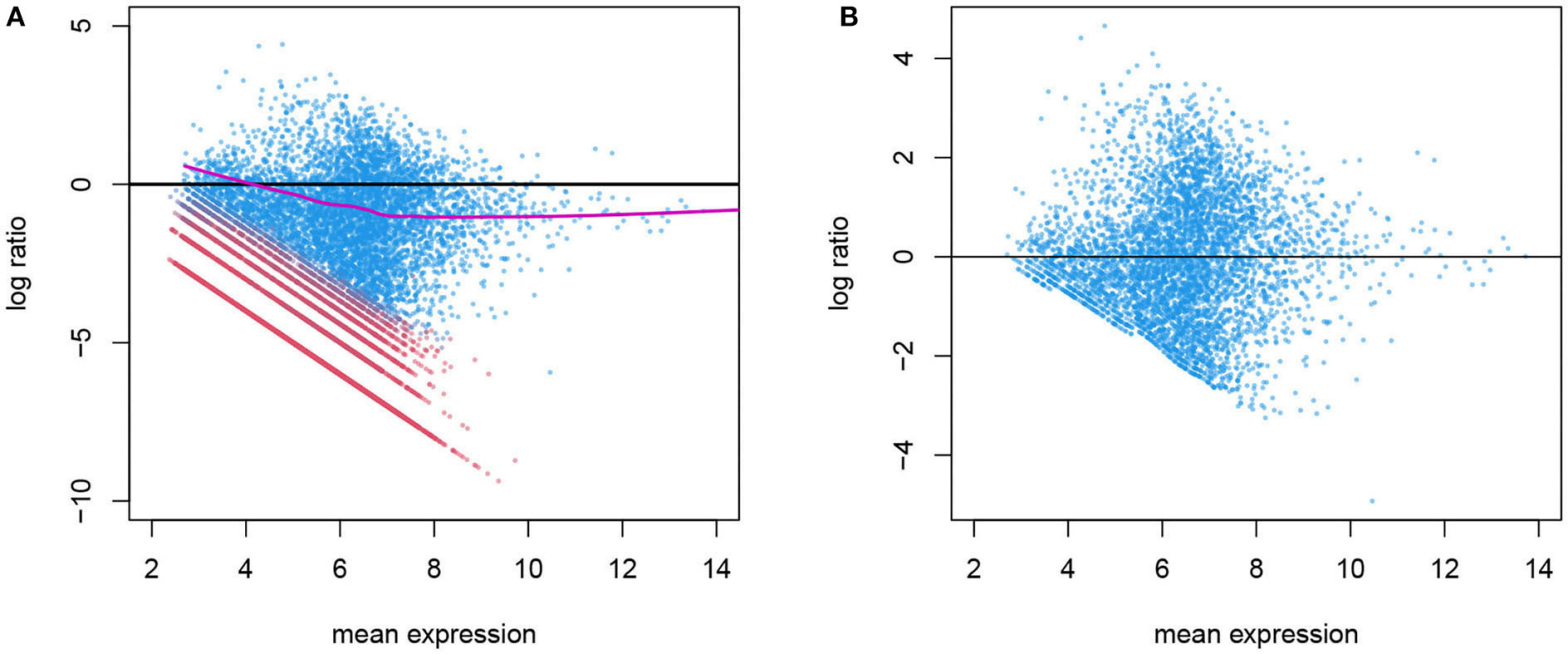
**(A)** Before normalization MA plot for gene counts before normalization. The log ratio of a gene's count in one cell over its mean expression level is plotted against the log mean expression (the alpha cells in the T2D data are used here). Genes in active expression phase are shown in blue and genes in background phase shown in red. Only the blue genes are used in estimating the systematic bias, shown in purple. **(B)** After normalization: the log ratio from normalized genes in the active phase shows no systematic bias.

The variation in gene counts is a combined result of biological variation, which we desire to retain, *systematic* technical variation, which we aim to remove in normalization, and lastly, random noise, which is not identifiable from the biological variation. In Figure 3, we illustrate an example of the systematic bias manifested differently in the two latent phases. This figure is similar to the “MA plot" commonly used in gene expression microarray data. Here, each point represents a gene. The x-axis is the mean expression within a given cell type, and the y-axis is the log ratio of a gene's count in this particular cell versus the mean expression level. This plot shows the overall pattern of bias as a function of expression level. A symmetrical scatter of points around the *y* = 0 line reflects no need for normalization. A simple linear effect of the library size leads to a constant bias in the log scale, hence the points shift vertically, and will be symmetrical around *y* = log *L*_*i*_ − log *L*_0_ for sample *i*, where *L*_*i*_ and *L*_0_ are the library sizes for the specific cell and the reference (typically set to be the median library size in a data set). However, sometimes the bias depends on the expression level and cannot be captured by one constant, and a non-linear normalization is needed. This has been used for diagnosis as well as for estimating and removing the systematic bias in microarray data (Bolstad et al., 2003). One key difference is that in scRNA-seq data, not all genes in a cell are affected by the systematic bias to the same extent. As shown in Figure 2, a gene's count is affected only when it is in the active phase. Thus, counts from genes who are in the background phase do not contain information about the sequencing efficiency, and should not be included in the estimation of the systematic bias.

In Wu et al. (2018) we show that the distribution of background counts and that of genes in the active phase are cell- and gene-specific, so a universal cutoff to determine the phase is not ideal. We describe a mixture model using a zero-inflated Poisson distribution and a lognormal-Poisson distribution for the two phases and estimate the conditional probability that a gene is in the active phase, given its gene identity and the cell context. This allows us to divide the counts in a cell to the two phases as shown in Figure 3. The systematic bias due to inconsistent sequencing efficiency can then be estimated as a smooth curve using the gene counts in the active phase alone.

### 2.5. Removing the Count-Depth Dependence

**Figure 4:**
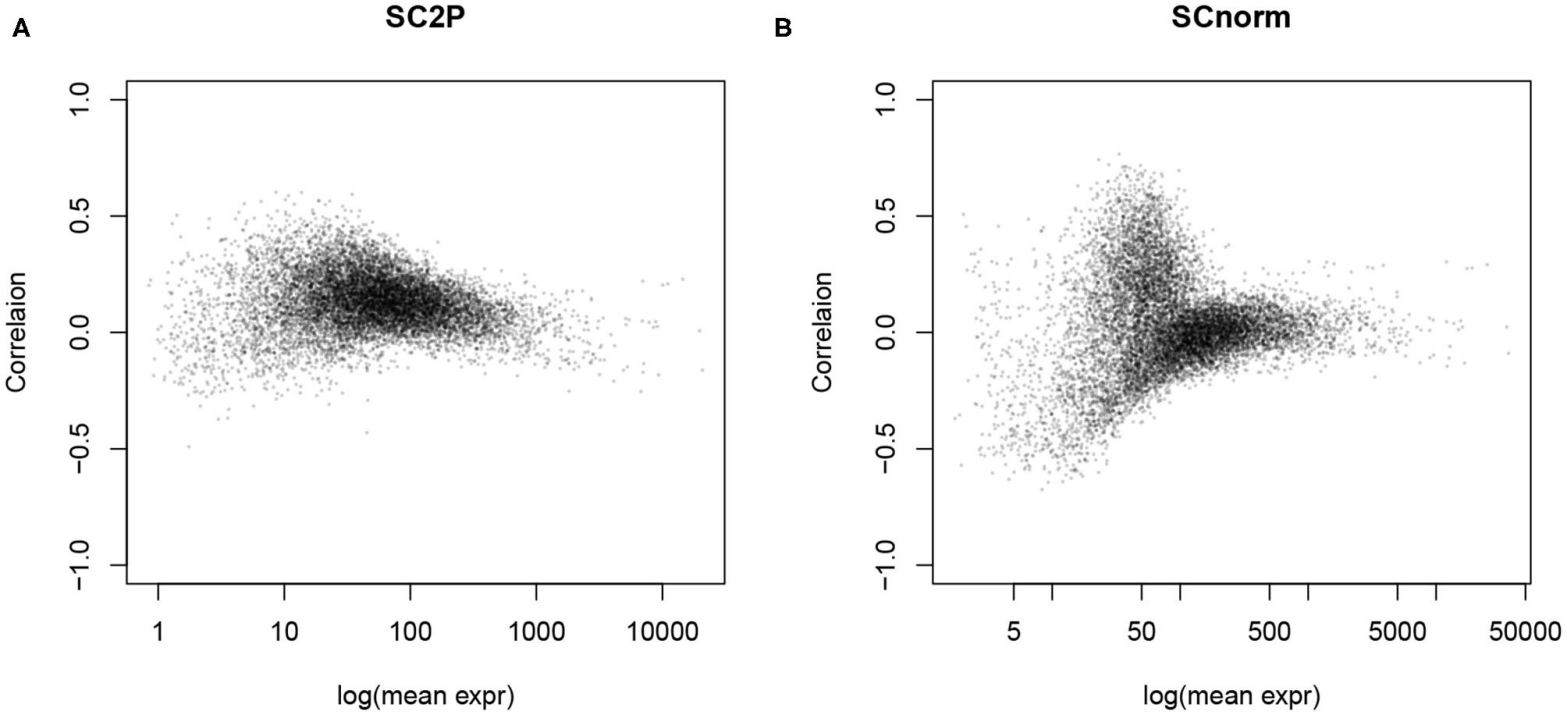
The count-depth relationship, measured as the correlation between the (normalized) gene counts and library size, is reduced after normalization. **(A)** Normalized by SC2P normalization. **(B)** Normalized by SCnorm.

The goal of normalization procedures is to remove technical variability without removing biological variability. One indication of unwanted technical variability is that gene counts are positively correlated with library size, referred to as the count-depth relationship (Supplementary Figure 2A). After adjusting for size factors, this strong correlation is often reduced toward zero, as seen in Figure 4 and Supplementary Figures 2B–D, since many normalization factors directly aim to remove the library size effect. However, we also notice that negative correlation is often introduced to genes with lower average expression levels in simple global normalization approaches, indicating an over-adjustment for those genes. SCnorm and SC2P both reach a near 0 correlation overall, with the result from SC2P closer to zero for genes over a wider range of mean expression level. Supplementary Figure 3 reveals the similarity and difference between SC2P and SCnorm more directly by plotting the raw and normalized counts in the same cell. We see that both methods adjust the higher counts even higher, but lower counts to a lesser extent. SCnorm partitions genes into several groups, each forming a curve, with different levels of adjustment. SC2P does the adjustment in a smooth fashion without putting genes in discrete categories, thus lacking apparent clusters in the figure.

### 2.6. Removing Technical Variation and Maintaining Biological Difference

**Figure 5:**
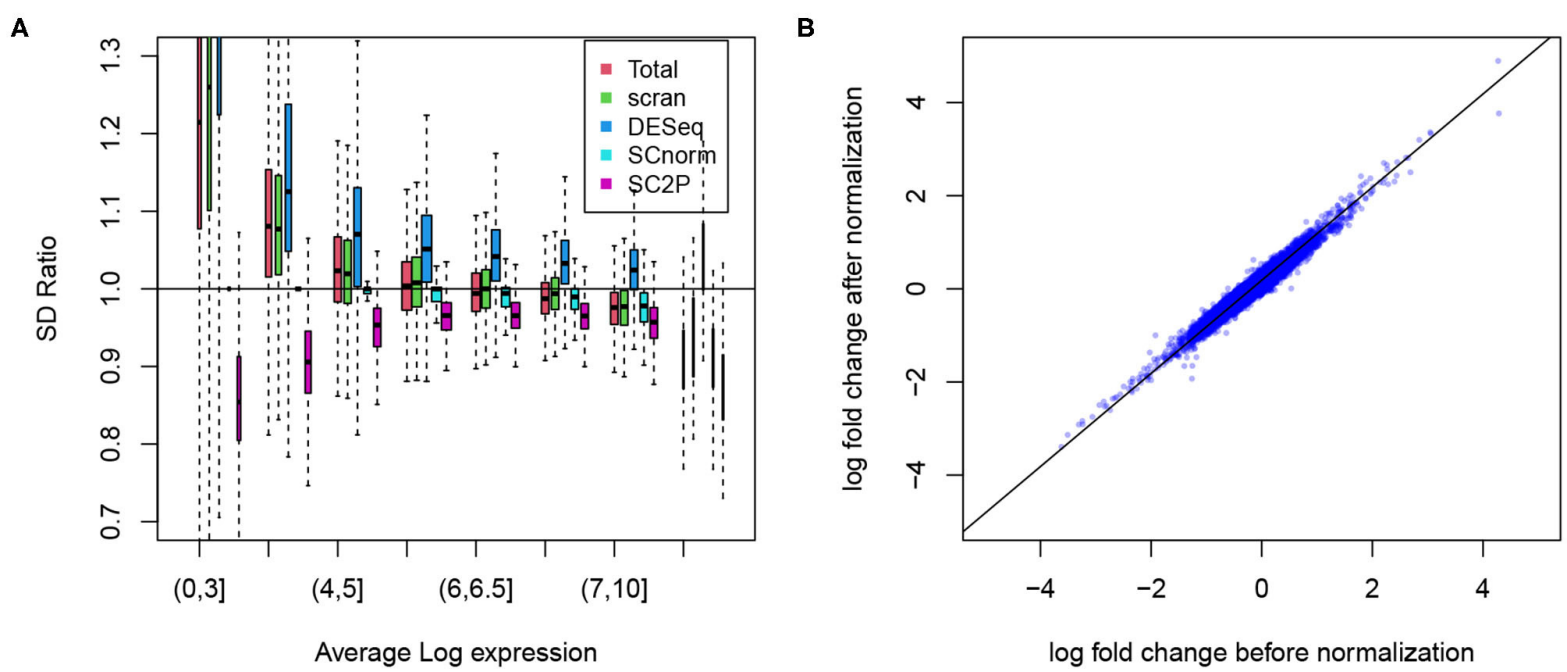
**(A)** Reduction of the technical variability among replicate cells. The ratio of gene specific standard deviation in normalized log counts over that in the raw log counts plotted. Genes are displayed in different groups based on their average expression when they are expressed. **(B)** The log fold change between Alpha and Beta cell populations before and after normalization remains at similar levels.

To show the success in removing technical variations, we first compare the conditional standard deviation of gene expression levels. Since dropout is a common phenomenon in scRNA-seq data, even strong cell type marker genes are not always observed in the corresponding cell type. Thus, marginal standard deviations often obscure the actual variability (Supplementary Figure 1). For each gene, we compute the standard deviation of its expression level when the gene is reliably detected, based on the posterior probability of a gene in the active phase. Among cells of the same type, we expect that the variance has sources of both biological and technical origins, and we expect that the variance reduces in normalized data. To evaluate the reduction in variance we compute the ratio of the variance in the normalized versus raw data. In Figure 5A we compare the ratio in genes stratified by average expression levels, in Alpha cells from the T2D data. Several methods (SCnorm, scran, and SC2P) can reduce the variance in highly expressed genes. Many, however, lead to an increase of variation for genes with lower expression levels. SC2P is the only method that can reduce the variance throughout the entire range of mean expression. In this particular data set, the normalization in DESeq actually increased the variance.

We certainly want to make sure that we do not reduce signal in the process of removing technical variation. To confirm this we show the difference in average expression between the Alpha and Beta cells. As shown in Figure 5B, the log fold change computed in SC2P normalized data maintains the between cell type differences. Similar results from the embryo data are included in the Supplementary Figure 4.

### 2.7. Removing Bias Due to Unbalanced Technical Bias

When the technical biases are randomly and evenly distributed in two cell populations, the population mean expression suffers from much smaller bias than the expression level in individual cells, since the law of large numbers will make the average of technical noise converge to zero when the number of cells increases. However, when two populations of cells in comparison have different distributions of technical effects, we may have biased result even in population means. For example, if one cell population tends to have more deeply sequenced cells than the other cell population, we will observe a bias in the mean expression levels, and DE observed across the two groups may simply reflect the imbalance in sequencing depth in the two populations. Successful normalization should remove such biases without introducing new biases.

**Figure 6:**
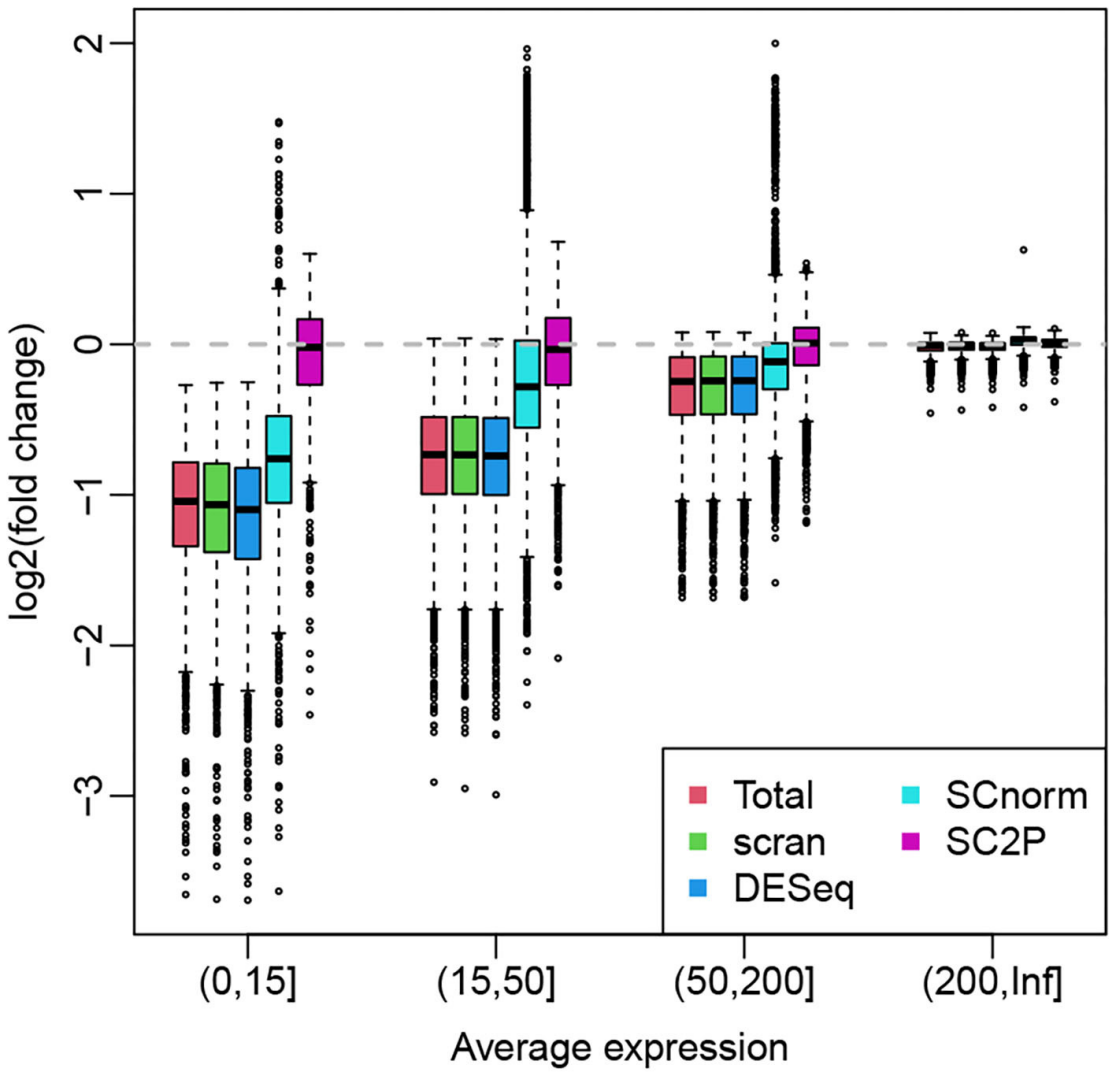
Box plots of log fold changes of normalized gene expression from different methods. The genes are stratified by average expressions. The log fold changes are computed based a two-group comparison, where the two groups contains cells with high and low sequencing depths. All cells are H1 hES cells.

For illustration purpose, we use the hESC data set that profiles H1 cells with both high and low sequencing depth so the systemic bias is obvious. When the sequencing depth is unbalanced between the two groups, the group with more highly sequenced cells tend to have average expression biased up, creating positive log fold change in genes without true DE. Here we compare the ability of various normalization methods in their ability to remove this potential bias. Figure 6 shows the boxplots of log fold changes of normalized gene expression for a two-group (the same type of cells in high- vs. low-sequencing depth groups) comparison, where the genes are stratified by average expressions. Since there is no biological difference between the two groups, we expect the log fold changes to be around zero. We see that, for highly expressed genes, all methods appear to remove the technical bias and show a median at zero. For lower expressed genes, the normalization methods using a cell-wize normalization factor (Total, scran, and DESeq) actually introduce biases to the data. This is because the lower expressed genes are affected by the library size in a lesser degree, thus they are over-normalized. SCnorm, by normalizing genes in different groups, can alleviate this problem to some extent and show smaller bias after normalization. SC2P is the only normalization that works well for genes with different average expression levels.

### 2.8. Impact on Downstream Analysis

The flexibility and single cell resolution of the scRNA-seq technology lead to a wide variety of applications and a large number of new analysis methods. To illustrate the consequences of normalization procedures on downstream analysis, we present two examples below. The first is differential expression analysis. Due to the lack of biological ground truth, we do not directly compare the accuracy of DE magnitude or the sensitivity of DE detection. Instead, we assess the impact of normalization on the robustness of DE detection. In scRNA-seq, the number of cells in each population is often orders of magnitude higher than the number of samples in most bulk RNA-seq data. A robust and reproducible analysis should not have results that are sensitive to the inclusion or removal of a few cells. We illustrate with the time course data and compare expression between time points We show that different normalization methods lead to different reproducibility in the time course data. When 5 cells, either the ones with the highest library size, or randomly chosen, are removed from the data set, our normalization shows much less disruption. In contrast, data normalized with other alternatives could lead to drastic changes (Supplementary Figure 5).

**Figure 7:**
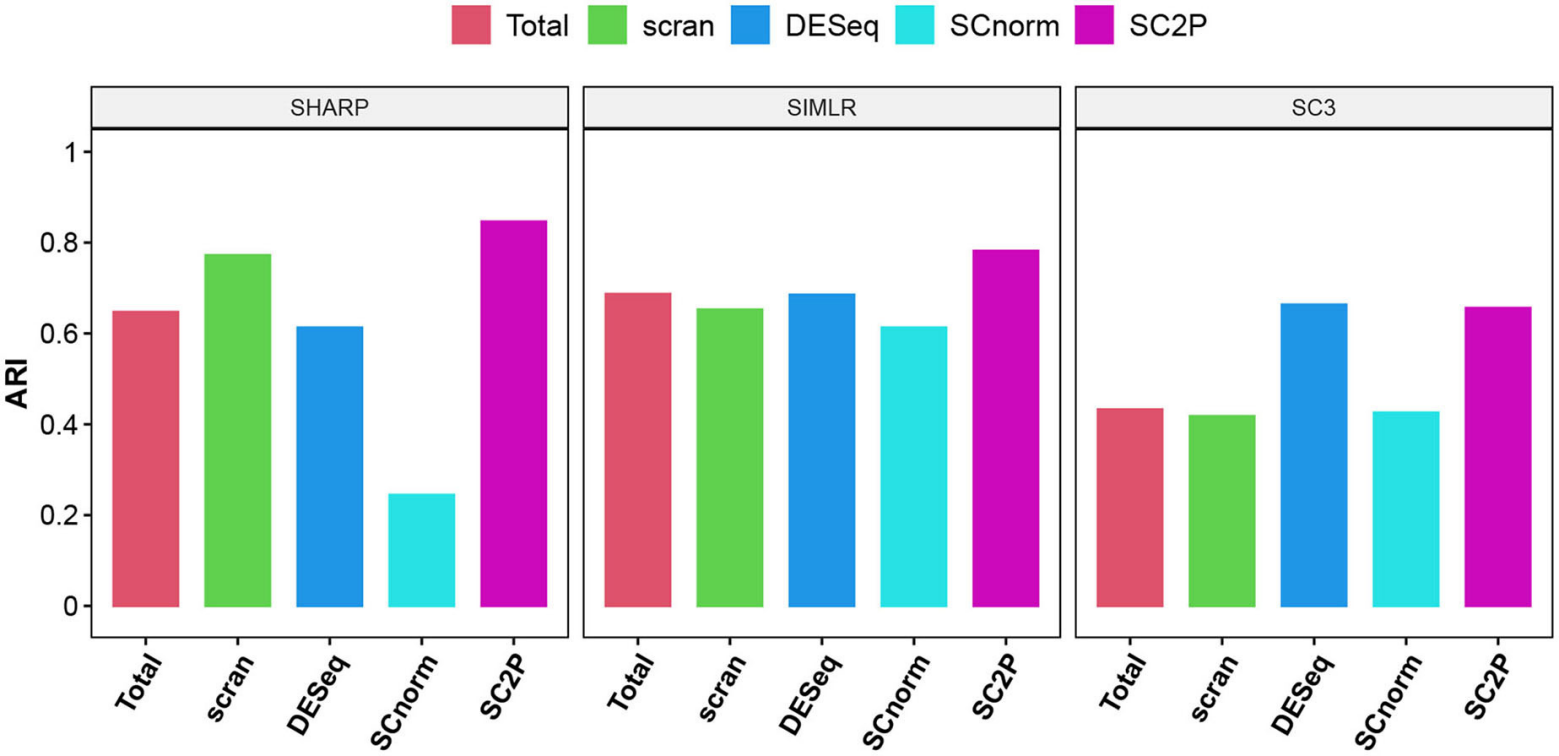
The adjusted Rand index (ARI) using three different clustering methods with different normalization. Genes that are detected in at least three cells are used in clustering.

We also compare the impact on clustering using the embryo data. We use log transformed pseudo counts after different normalization in three widely used scRNA-seq clustering methods, including SIMLR ((Wang et al., 2017), SHARP (Wan et al., 2020), and SC3 Kiselev et al. (2017). Figure 7 compares the Adjusted Rand Index (Hubert and Arabie, 1985), which measures the concordance of pair-wise relationship between each pair of cells with known developmental stages, adjusted for the agreement due to coincidence. The proposed normalization has the highest ARI in all three methods.

## 3. Discussion

We present a normalization method that provides a cell- and gene-specific normalization factor that borrows information across genes and across cells. Both the cell context and gene context are used in predicting whether a gene appears to be in the active phase in a given cell, and only the active ones are used in estimating the technical bias due to RNA extraction/amplification/sequencing. It is more flexible than simple size factor normalization, which adjusts all genes in a cell in a universal manner, but is still robust for the normalization is estimated from a large number of genes using only a few degrees of freedom.

scRNA-seq opens the door to many new applications beyond what is offered by bulk RNA-seq. It allows the query of the heterogeneity of individual cells, instead of the average of many. This means higher variability of the direct measurements, since the quantity measured is no longer a population average which is stabilized when millions of cells are pooled together. This often means that we have many more cells sequenced in an experiment, thus many more “samples" to work with. Compared to typical bulk RNA-seq data, the number of samples in a scRNAseq data is typically orders of magnitude higher. If differential expression (DE) between two populations of cells is of interest, and a gene-specific “count-depth relationship" confounds the DE, one may argue that we no longer need normalization before analysis. One could choose to adjust for this confounding in the regression setting, as is done in MAST (Finak et al., 2015). In a regression with sample size over several hundred, adding the library size as a covariate simply means using one degree of freedom to account for the “count-depth relationship." Since the regression is done for each gene, this allows gene specific adjustment. The drawback is that this assumes a linear effect of the library size, which may not be valid in all cells, and it can be sensitive to which cells are included in the analysis. This is also limited to the DE analysis, whereas scRNA-seq is used for many more applications.

This paper addresses normalization for scRNA-seq data in relatively high library size, without the use of unique molecular identifiers (UMI). When UMIs are used, the amplification bias is largely eliminated because multiple amplified copies of the same transcript is only counted once. These data sets still have a need for normalization because library size remains an obvious factor in the observed counts. But it is a different problem and beyond the scope of this manuscript.

## 4. Methods

### 4.1. Probability Model

We consider each gene in any given cell is either actively transcribed or not expressed. When it is transcribed (we refer to this as Phase II or the active phase), its expression level is represented as a concentration *θ*_*gi*_ for gene *g* in cell *i*. When it is not transcribed (we referred to this as the background phase), its count depends on a sample(cell)-specific noise distribution. As described in Wu et al. (2018), we model a gene's true expected concentration as a lognormal random variable, and the background noise as a zero-inflated Poisson (ZIP) distribution. The sequencing technology does not directly measure *θ*_*gi*_, because the RNA molecules in the cells have to be captured, reversed transcribed, amplified and eventually counted. To account for the potentially unequal counting efficiency for the RNAs of different genes in different cells, we use *S*_*gi*_ to represent the technical distortion for gene *g* in cell *i*.

The observed count thus comes from a mixture distribution with latent phase *Z*_*gi*_, where *Z*_*gi*_ = 1 means the gene is in the active phase. Thus, we have

$$\begin{array}{l} Y_{gi}|Z_{gi}=1,\theta_{gi}∼Poisson\left(\theta_{gi}S_{gi}\right)with\theta_{gi}∼LN\left(\mu_{g},\sigma_{g}^{2}\right),\\ Y_{gi}|Z_{gi}=0∼ZIP\left(p_{0i},{\text{λ}}_{i}\right) \end{array}$$

The parameters *θ*_*gi*_ and *S*_*gi*_ cannot be both uniquely identified. For identifiability we constraint the average of *S*_*gi*_ for the cell with the median sequencing depth to be 1. In Supplementary Figure 4 we show the observed log counts for a few example genes in the T2D data to illustrate that the normal assumption is a reasonable one for the active phase.

### 4.2. Estimating the Parameters

In Wu et al. (2018) we provide the details of the estimating procedures for obtaining the ${\hat{\mu }}_{g},{\hat{\sigma }}_{g}^{2}$ and ${\hat{p}}_{0},\hat{\lambda }$. We describe it briefly here. The ZIP parameters are estimated based on the properly of a linear relationship in the log frequency of Poisson counts, with the slope dependent on $\hat{\lambda }$. Thus, we can view the distribution of counts as ZIP contaminated by Phase II observations. We use a robust regression to down-weight the influence of high counts to obtain a robust estimate of λ and then use the amount of excessive zero to estimate *p*_0_. The initial phase indicators *Z*_*gi*_ are set based on the point mass from the ZIP model for each observation. The parameters *μ*_*g*_ and σ_*g*_ are then estimated using the counts in the active phase for each gene. This is iterated using the EM algorithm, which allows us to obtain a *Ẑ*_*gi*_ for each gene in each cell as well as ${\hat{\mu }}_{g}$.

### 4.3. Estimating the Normalization Factor

With these parameters we obtain residuals ${\hat{\epsilon }}_{gi}=\log Y_{gi}-{\hat{\mu }}_{g}$ for the genes deemed in the active phase (we use *Ẑ*_*gi*_ > 0.99), which has expectation log *S*_*gi*_ for each gene. Figure 3A shows an example of the distribution of the residuals against ${\hat{\mu }}_{g}$. When there is no need for normalization, ${\hat{\epsilon }}_{gi}$ shall be symmetrically distributed around the *y* = 0 line. When there is a consistent bias for all genes in the same cell, log *S*_*gi*_ ≡ log *S*_*i*_, ${\hat{\epsilon }}_{gi}$ may have a non-zero expectation but will show a common trend for all expression levels. However, in general, the bias is often related to the mean expression level, as shown in Figure 3A. We use a spline function to estimate a smooth relationship between *S*_*gi*_ and *μ*_*g*_, and obtain ${\hat{f}}_{i}$. This allows us to address the unequal need for normalization for different genes without having to put them in discrete categories. Then given a gene we estimate $\log S_{gi}={\hat{f}}_{i}\left(\log Y_{gi}\right)$.

A critical step here is to identify the genes in the active phase in a cell, as only these genes reflect the technical biases in mRNA extraction and amplification. Thus, in Figure 3A the smooth line is estimated using only the active phase genes (blue) only. Note that what we need is a good estimate for this curve, and thousands of genes in the active phase jointly determine this curve. Therefore, even if for any specific gene the phase determination may not be accurate, its influence on the curve is trivial.

### 4.4. Use of the Normalization Factor

The normalization factor has the interpretation of the potential detection bias for gene *g* in cell *i* if gene *g* is in the active phase. This value is irrelevant in the case that the gene is not active in a cell. Directly adjusting the raw counts indiscriminately, such as in TPM, often leads to inflation of gene counts in cells with low total counts, which may create misleading large fold changes across cells. Thus, we provide the normalization factor as an offset that can be incorporated into analysis pipelines that use the count data directly. To use the normalization factor for direct adjustment, we recommend filtering genes to focus on the ones that are actively expressed.

## Data Availability Statement

The datasets used for this study can be found in the Gene Expression Omnibus (GEO) under accession numbers GSE86473, GSE85917, GSE45719, and GSE75748. The method is implemented in the R package SC2P and available at https://github.com/haowulab/SC2P.

## Author Contributions

ZW conceived the method. HW contributed in the development, implementation, and evaluation. KS conducted the assessment of clustering analysis. All authors contributed to the article and approved the submitted version.

## Conflict of Interest

The authors declare that the research was conducted in the absence of any commercial or financial relationships that could be construed as a potential conflict of interest.
