# Supplementary material for: Non-linear Normalization for Non-UMI Single Cell RNA-Seq
Source: Front Genet. 2021 Apr 9;12:612670. doi: 10.3389/fgene.2021.612670 (PMC8063035; doi:10.3389/fgene.2021.612670)
Supplement: Supplementary file 1 [file Data_Sheet_1.PDF]

Supplementary Material

1 ZERO COUNTS ARE COMMON EVEN IN CELL TYPE MARKER GENES

In measuring a gene's variation in expression level when it is being actively transcribed, the simple variance may be inflating the actual variance because a gene may not be expressed in all the cells. This is true even for cell type marker genes that are expected to be highly expressed for a given cell type. The figures below show the gene ENSG00000141433 in alpha and beta cells. ENSG00000141433 is the gene with symbol *ADCYAP1*, which encodes Adenylate Cyclase Activating Polypeptide 1, a protein that incudes insulin secretion by beta cells. It is clearly highly expressed in most beta cells, but can still get zero counts. ENSG00000078098(FAP) is highly expressed in alpha cells in general and also missing from many alpha cell. The marginal variance within a cell type will reflect both the proportion of cells that the gene has no counts and the variation of the magnitude of expression when the gene is expressed.

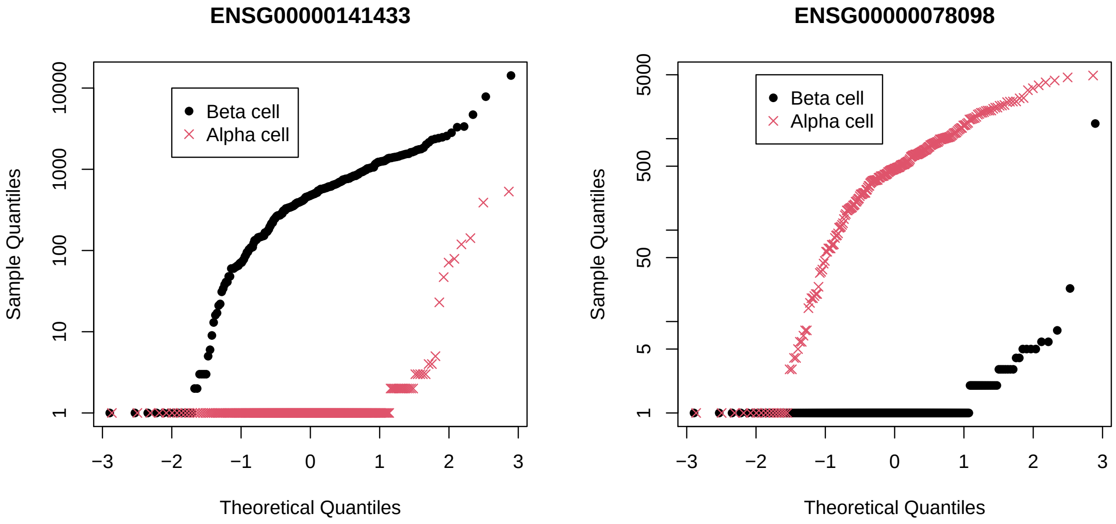

Figure S1: Quantile-quantile plot of gene counts (counts +1, to allow display in log scale) of the ENSG00000141433 (ADCYAP1) gene and ENSG00000078098(FAP) in alpha and beta cells

2 COUNT-DEPENDENCE BEFORE AND AFTER NORMALIZATION

Library size reflects the combined technical effect on all genes due to mRNA extraction, amplification efficiency and sequencing depth. If these technical factors impact genes in the similar way, the combined effects do not cancel out and show at the cell level. Thus we often observe a strong correlation between library size and gene counts, especially for genes that are expressed at higher values and less influenced by Poisson counting error. The figures below shows conditional correlation between gene counts and library size when counts are above 30. Normalization methods that aim to adjust for a cell-wise global factor often removes this relationship for highly expressed gene, but over-adjust the genes that are expressed at lower levels. As a result, the correlation with library size reduces near 0 for high expression genes, but a negative correlation is introduced among genes that have modest mean expression.

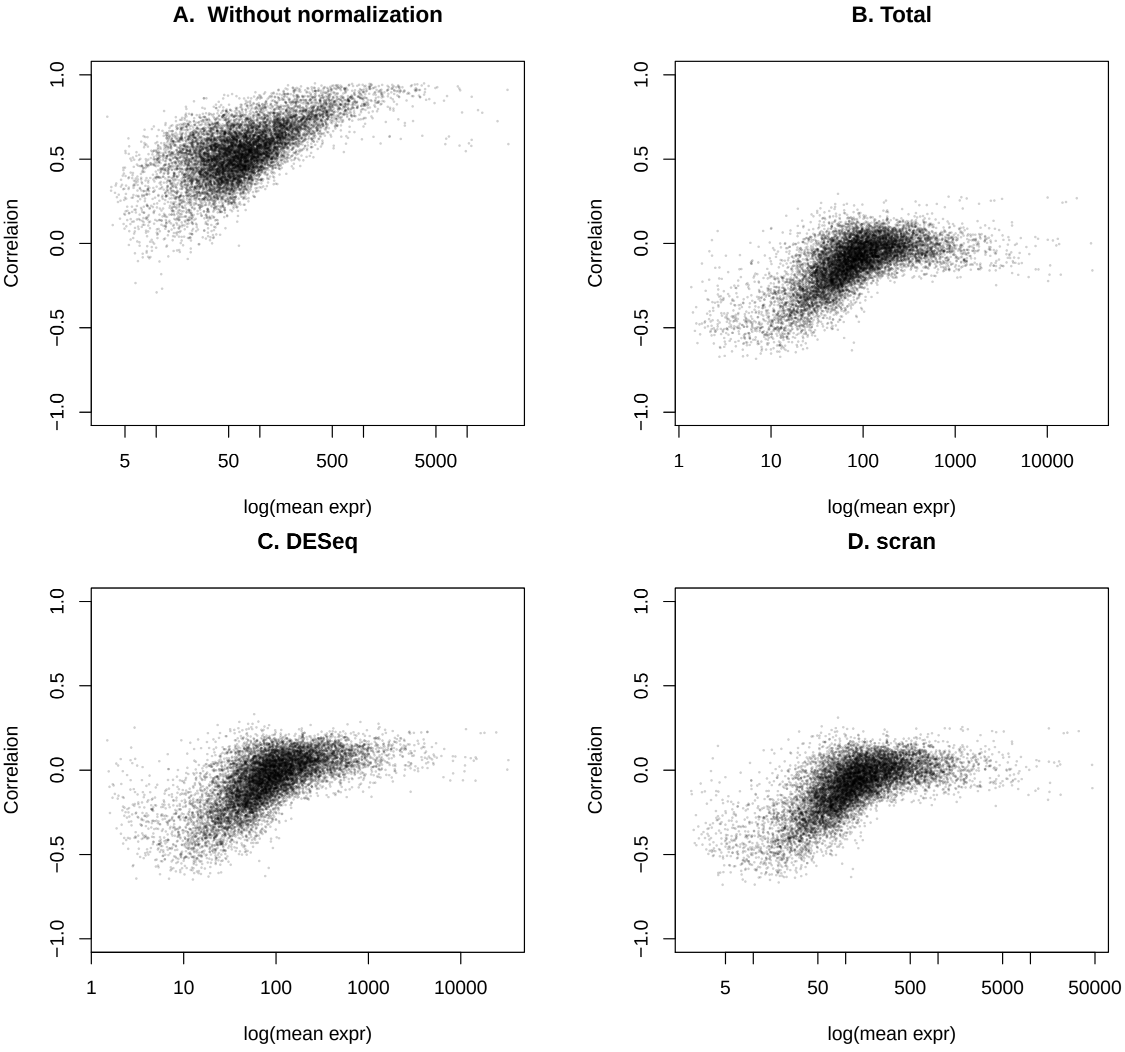

Figure S2: Count-depth relationship before and after normalization. Correlation between gene counts and library size is strong before normalization and reduced in normalized data. Total: normalized data are counts per million (CPM).

3 SC2P AND SCNORM: SMOOTH NONLINEAR NORMALIZATION VERSUS GENE GROUPS

SCnorm puts genes in several groups based on their mean expression and allows separate normalization for each group. This is reflected by the discrete lines seen in the before and after normalization. SC2P does a smooth nonlinear normalization without categorizing the genes. As a result we see a similar before/after relationship, but with a smooth transition.

In this particular example, genes with higher counts are typically further away from the identity line, indicating a greater level of adjustment. Genes with lower counts are adjusted at various levles, some much closer to the identity line, suggesting that little normalization is done to these genes in this cell.

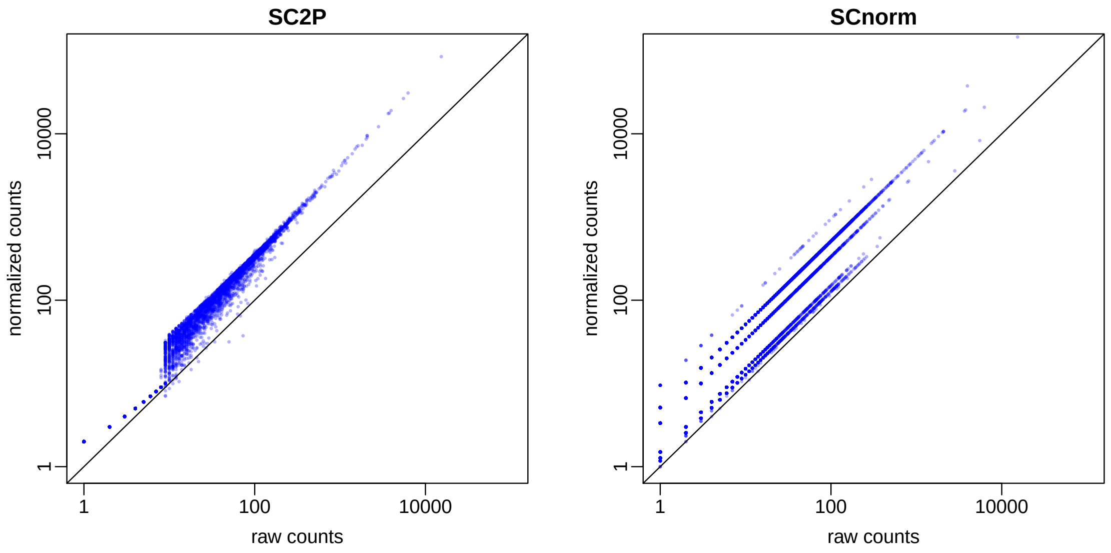

Figure S3: Normalized counts versus raw counts in one example cell. The black straight line is the identity $y = x$ line.

4 ADDITIONAL RESULTS ON REMOVING TECHNICAL VARIATION AND MAINTAINING BIOLOGICAL DIFFERENCE

As in Section 2.6 in the main text, we evaluate the reduction in variance by computing the ratio of the variance in the normalized versus raw data. We confirm that biological difference is not reduced by comparing the log fold change between biological groups. Figure S**??** shows the same comparisons from the embryo data.

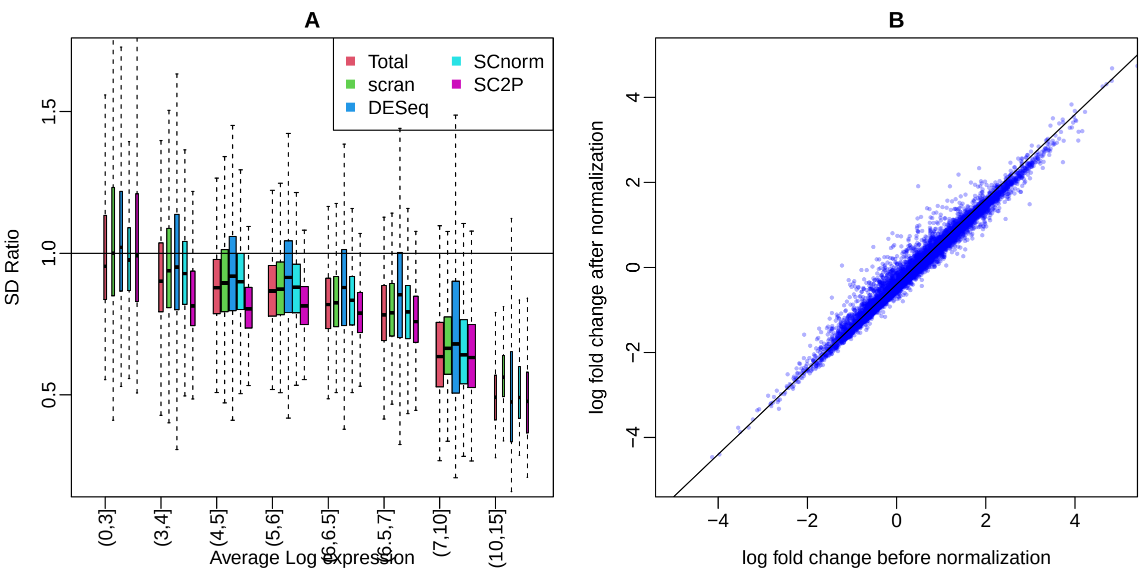

Figure S4: A) Reduction of the technical variability among replicate cells. The ratio of gene specific standard deviation in normalized log counts over that in the raw log counts plotted. Genes are displayed in different groups based on their average expression when they are expressed. B) The log fold change between cells from the four-cell and eight-cell stages, before and after normalization.

5 REPRODUCIBILITY IN DE ANALYSIS

We use the time course data to illustrate the reproducibility of differential expression detection. For comparison we use MAST instead of the SC2P DE detection to analyze all normalized data. Since the normalization is adjustment of the gene expression level, the direct impact is on the detection of the DE in the continuous component instead of the discrete component. We compare the p-value testing the DE between 12 hour and 24 hour using all cells with two perturbations. We delete five cells with the highest library size, or five cells randomly. With a total of 168 cells, the deletion of 5 cells should not have a large effect on the statistical power. A robust analysis would yield similar p-values. Figure SS5 shows the comparison of p-values of DE analysis between the dataset with cells removed versus the full data with different normalization. The results in SC2P normalization is the most reproducible, and with fewer genes with qualitatively different inference (p-value close to 1 in one analysis and very significant in another).

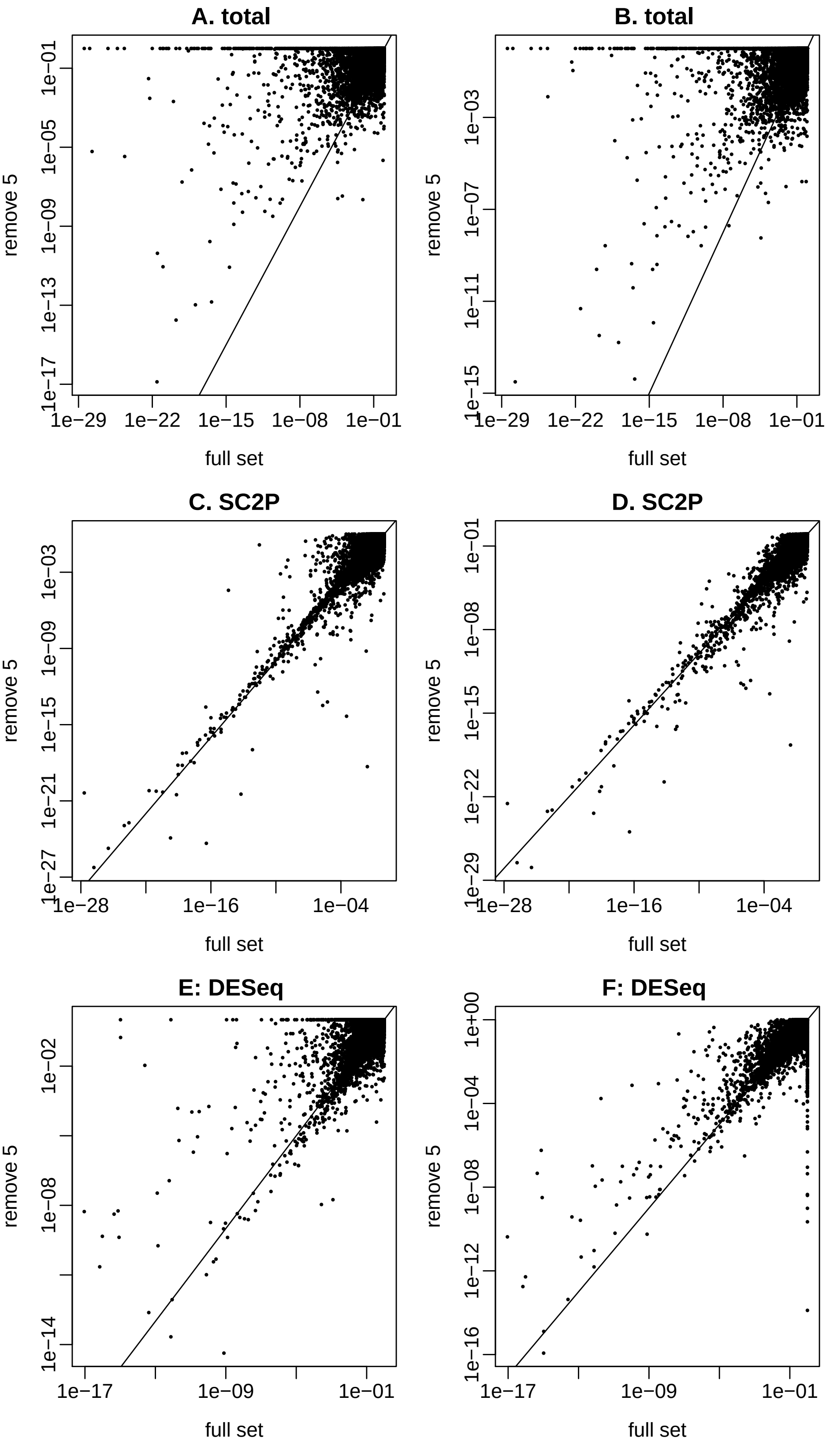

Figure S5: Robustness of DE analysis. DE analysis between cells at 12 hour and 24 hour in the time course data are performed with either all cells, or 5 cells removed. A,C,E: The 5 cells removed are the ones with the highest library size. B,D,F: The 5 cells removed are randomly chosen.

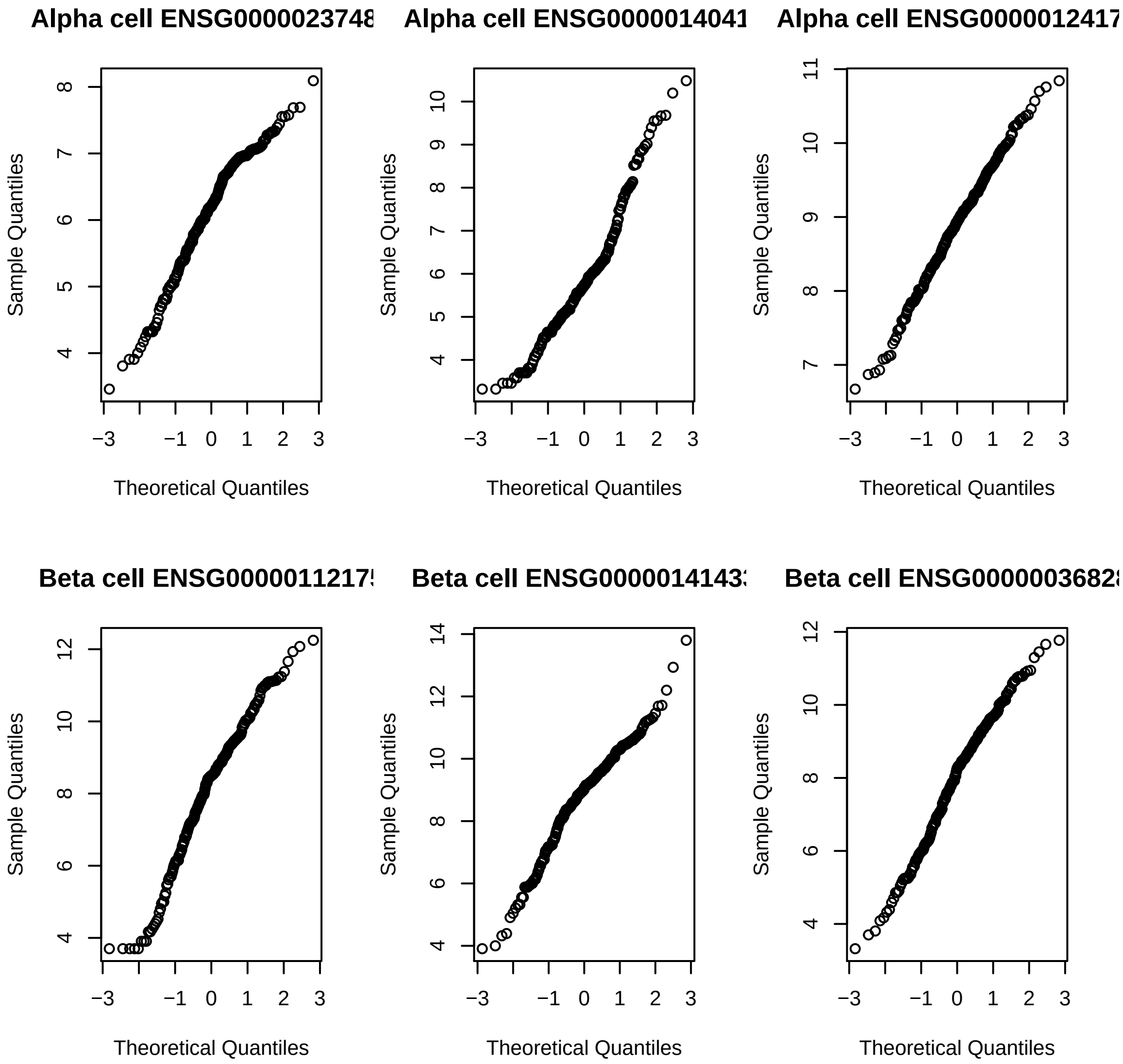

Figure S6: Quantile-quantile plot of gene counts in the active phase

6 LOGNORMAL ASSUMPTION ON EXPRESSION LEVEL

A few examples of the gene counts across cells when the gene is actively transcribed. Quantile-quantile plots for genes in alpha and beta cells in the T2D data are shown.
